# Simulation of New York City’s Ventilator Allocation Guideline During the Spring 2020 COVID-19 Surge

**DOI:** 10.1001/jamanetworkopen.2023.36736

**Published:** 2023-10-05

**Authors:** B. Corbett Walsh, Jianan Zhu, Yang Feng, Kenneth A. Berkowitz, Rebecca A. Betensky, Mark E. Nunnally, Deepak R. Pradhan

**Affiliations:** 1 Division of Pulmonary, Critical Care, and Sleep Medicine, Department of Medicine, New York University Grossman School of Medicine, New York; 2 Division of Pulmonary, Critical Care, and Sleep Medicine, Department of Medicine, University of Los Angeles David Geffen School of Medicine, Los Angeles; 3 Section of Palliative Medicine, Department of Medicine, University of Los Angeles David Geffen School of Medicine, Los Angeles; 4 Department of Biostatistics, New York University School of Global Public Health, New York; 5 National Center for Ethics in Health Care, Veterans Health Administration; 6 Division of Medical Ethics, Department of Population Health, New York University Grossman School of Medicine, New York; 7 New York University Langone Health, New York; 8 Department of Anesthesiology, Perioperative Care, and Pain Medicine, New York University Grossman School of Medicine, New York; 9 Bellevue Hospital Center, NYC Health & Hospitals, New York, New York

## Abstract

**Importance:**

The spring 2020 surge of COVID-19 unprecedentedly strained ventilator supply in New York City, with many hospitals nearly exhausting available ventilators and subsequently seriously considering enacting crisis standards of care and implementing New York State Ventilator Allocation Guidelines (NYVAG). However, there is little evidence as to how NYVAG would perform if implemented.

**Objectives:**

To evaluate the performance and potential improvement of NYVAG during a surge of patients with respect to the length of rationing, overall mortality, and worsening health disparities.

**Design, Setting, and Participants:**

This cohort study included intubated patients in a single health system in New York City from March through July 2020. A total of 20 000 simulations were conducted of ventilator triage (10 000 following NYVAG and 10 000 following a proposed improved NYVAG) during a crisis period, defined as the point at which the prepandemic ventilator supply was 95% utilized.

**Exposures:**

The NYVAG protocol for triage ventilators.

**Main Outcomes and Measures:**

Comparison of observed survival rates with simulations of scenarios requiring NYVAG ventilator rationing.

**Results:**

The total cohort included 1671 patients; of these, 674 intubated patients (mean [SD] age, 63.7 [13.8] years; 465 male [69.9%]) were included in the crisis period, with 571 (84.7%) testing positive for COVID-19. Simulated ventilator rationing occurred for 163.9 patients over 15.0 days, 44.4% (95% CI, 38.3%-50.0%) of whom would have survived if provided a ventilator while only 34.8% (95% CI, 28.5%-40.0%) of those newly intubated patients receiving a reallocated ventilator survived. While triage categorization at the time of intubation exhibited partial prognostic differentiation, 94.8% of all ventilator rationing occurred after a time trial. Within this subset, 43.1% were intubated for 7 or more days with a favorable SOFA score that had not improved. An estimated 60.6% of these patients would have survived if sustained on a ventilator. Revising triage subcategorization, proposed improved NYVAG, would have improved this alarming ventilator allocation inefficiency (25.3% [95% CI, 22.1%-28.4%] of those selected for ventilator rationing would have survived if provided a ventilator). NYVAG ventilator rationing did not exacerbate existing health disparities.

**Conclusions and Relevance:**

In this cohort study of intubated patients experiencing simulated ventilator rationing during the apex of the New York City COVID-19 2020 surge, NYVAG diverted ventilators from patients with a higher chance of survival to those with a lower chance of survival. Future efforts should be focused on triage subcategorization, which improved this triage inefficiency, and ventilator rationing after a time trial, when most ventilator rationing occurred.

## Key Points

**Question:**

How would implementation of the New York Ventilator Allocation Guidelines (NYVAG) have affected overall mortality and health disparities during the apex of the COVID-19 2020 surge?

**Findings:**

In this cohort study of 674 intubated patients, simulated ventilator rationing occurred for nearly 1 in 4 patients over 15 days. While NYVAG did not exacerbate existing health disparities, 44.4% of those simulated for ventilator rationing would have survived if provided or maintained on a ventilator while only 34.8% of those newly intubated patients receiving a reallocated ventilator survived.

**Meaning:**

These results suggest that NYVAG, as written, might unintentionally divert ventilators to those with a lower chance of survival.

## Teaser

This cohort study simulates the outcomes of implementation of the New York State Ventilator Allocation Guidelines during the spring 2020 surge of COVID-19 at a single health system in New York City.

## Introduction

The COVID-19 pandemic triggered unprecedented strain on the health care system. During the spring 2020 surge in New York City (NYC) there was a palpable concern that ventilators might need to be rationed by implementing crisis standards of care (CSC).^1,2,3,4^ Many CSC provide a protocol for triage whereby patients are ordered to potentially receive a scarce resource. Such a triage process, with the magnitude of supply limitation, determines which patients receive that resource. This consequentially determines both the decision to allocate (to provide or withhold), as well as to potentially reallocate (to maintain or withdraw) scarce resources. We define a scarce resource to be *rationed* when it is withheld or withdrawn from one individual so it can be provided to another individual. One case of ventilator rationing occurred during the spring 2020 COVID-19 surge in NYC.^5^ This case followed the New York Ventilator Allocation Guidelines (NYVAG),^6^ refined in 2015 to draw upon a community’s duty to care, resource stewardship, transparency, and distributive justice to save the most lives. This unvalidated guideline centers on the Sequential Organ Failure Assessment (SOFA) score, a summative score on a 24-point scale of organ dysfunction across neurologic, pulmonary, cardiovascular, hematologic, hepatobiliary, and renal subscores intended to guide ventilator triage. NYVAG dictates patients to be triaged and potentially provided a ventilator for a prespecified duration of time. After this time trial, their SOFA score is recalculated and patients are retriaged to maintain their ventilator or have it reallocated to another patient. While the overwhelming majority of CSC utilize a SOFA score,^7,8,9,10^ concerns remain about its ability to predict mortality^11^ or the possibility it might worsen existing social disparities.^12,13,14,15^ Despite these potential shortcomings, NYVAG is operationally similar to many other current guidelines.^7,9,16,17,18^

While other studies may have simulated NYVAG^19,20,21,22,23,24,25,26,27,28^ or other CSC,^29,30,31,32^ most perform a limited analysis of how resources might be allocated to patients not already receiving that resource (front-end triage) and do not consider potential reallocation after a time trial (back-end triage). Triage, a critical component of CSC, includes both front-end and back-end triage. One study^33^ appears to simulate a modified NYVAG protocol but it only provides limited insight into the aggregated performance and without attention to front-end and back-end triage. The authors instead focus on varying simulation assumptions (ventilator capacity and probability of death of those experiencing rationing) and comparing the aggregated performance with a first-come, first-served strategy. This adds to the understanding of variable conditions of NYVAG implementation but does not evaluate for NYVAG triage inefficiencies resulting in unintentional prioritization of patients at increased risk of mortality. Accordingly, the focus of this study is to perform a simulated descriptive analysis, under realistic resource limitations and pandemic strain, to assess how NYVAG might have performed with a focus on triage-related deaths and health disparities. We also aimed to identify improvements in NYVAG that could potentially improve triage inefficiencies and lead to improved mortality outcomes.

## Methods

Adult patient medical records were retrospectively reviewed from a single academic hospital system from March 1, 2020, to July 1, 2020 (ie, during the surge), and included if they were ventilated. The hospital system had 263 prepandemic ventilators excluding anesthesia machines.^34^ In this simulation study, a crisis period requiring ventilator triage per NYVAG was defined as once ventilator demand exceeded 95% prepandemic ventilators (250 patients).^35^ Patients’ SOFA scores were queried from the health record while ventilated, with missing SOFA subscores presumed normal (ie, scored as 0).^19,22,27,36^ Race and ethnicity were included in study data because of growing concern that existing crisis standards of care protocols might worsen existing social disparities; data were based on self-report by the patient or identified unknown if unable or unwilling to self-identify.

This study followed the Strengthening and Reporting of Observational Studies in Epidemiology (STROBE) reporting guideline. It was approved by NYU School of Medicine’s institutional review board with a waiver of informed consent because it was a retrospective study of medical records.

**Figure 1.**
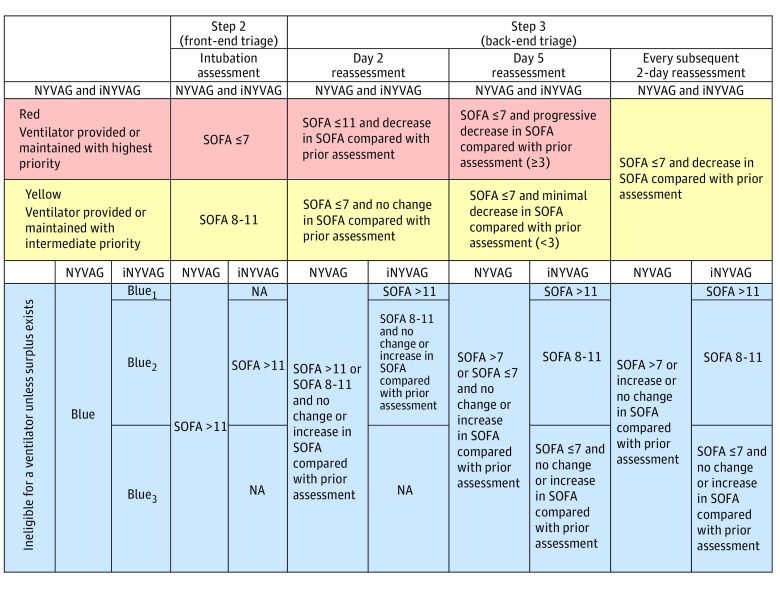
New York State Ventilator Allocation Guidelines (NYVAG) and Improved Guidelines (iNYVAG) Steps 2 and 3 There were 3 gaps in the original NYVAG that were addressed as follows: (1) patients with a SOFA score of 7 on the day of consideration for intubation were categorized as red; (2) individuals with a SOFA score greater than 7 on any assessment after 5 days of mechanical ventilation were ineligible for a ventilator; (3) patients ventilated for 7 or more days who continued to have a SOFA score ≤7 and decreasing were placed in the yellow category. The improvements to the NYVAG model made by iNYVAG included subcategorization of the blue category into 3 groups (Blue_1_-Blue_3_) to incorporate the acuity of illness on the day of evaluation. NA indicates not applicable; SOFA, Sequential Organ Failure Assessment.

The simulation model used ventilator triage as described by NYVAG^6^ (Figure 1; eMethods in Supplement 1). Step 1, the application of exclusionary criteria, was satisfied from a medical record review (eFigure 1 in Supplement 1). Step 2 is the consideration for intubation and potential allocation of a ventilator (front-end triage). Step 3 is the potential reallocation of a ventilator after a prespecified time trial (back-end triage). Simulated ventilator rationing occurred by exclusion during step 1, failure to allocate during step 2, or reallocation during step 3 and were simulated to expire in a manner similar to several previous studies.^20,28,29,33^ NYVAG prioritizes patients to receive or maintain a ventilator in a color-coded system, as red (high), yellow (intermediate), or blue (only provided a ventilator if a surplus exists).^37^ On any day during the simulated crisis period, ventilators were first provided at random to those individuals not due to be reassessed (ie, those that were receiving a ventilatory time trial), then randomly to patients categorized as red, then randomly to those patients classified as yellow, and lastly randomly to patients triaged as blue. In cases of inadequate ventilator supply for all individuals in a priority category, employing randomization as a secondary triage strategy might lead to the selection of different individuals for ventilator rationing, thereby influencing subsequent rationing and simulated outcomes during the crisis period. A total of 20 000 simulations of the surge (10 000 NYVAG and 10 000 of the improved NYVAG guidelines [iNYVAG]) were performed in R Core Team version 4.3.1 to account for this randomization. A proposed improvement NYVAG (iNYVAG), whereby the blue category was further subcategorized by SOFA score on the day of evaluation, was made and its performance simulated (Figure 1). The identification and distribution of available ventilators, those originating from individuals extubated, deceased, or placed on extracorporeal membrane oxygenation the previous day are described in eMethods in Supplement 1.

### Statistical Analysis

Outcome end points included both observed and simulated (hypothetical in the simulation) outcomes. Because every patient in our cohort received a ventilator in a clinical setting, their survival concerning hospital discharge with continued ventilation was known (primary observed end point). Secondary observed end points included, for those patients experiencing front-end triage, triage category by demographics and survival to discharge. The primary simulated end point was the number of patients that had their ventilators rationed during the simulation. Secondary simulated end points included, for those patients whose ventilator was rationed, demographics, day of mechanical ventilation the ventilator was rationed, and SOFA score trajectory during the time trial. Analysis was completed in R version 4.3.1 (R Project for Statistical Computing) and Excel 2022 (Microsoft). Categorical data were analyzed with the χ^2^ test using the 95% CI. Continuous variables were compared with a 2-tailed *t* test using 5% significance level.

## Results

**Figure 2.**
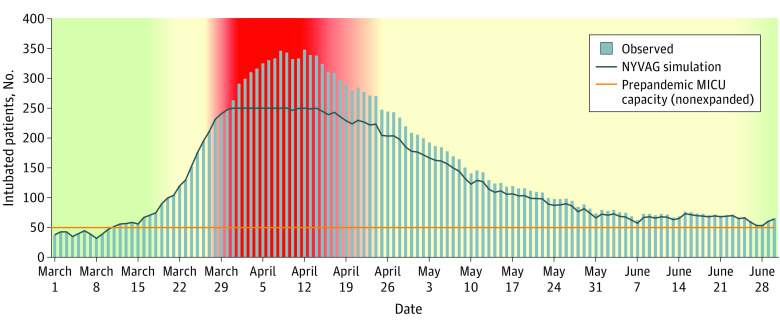
Total Intubated Patients Throughout the Spring 2020 COVID-19 Surge in a Single New York City Hospital System The red shading represents the crisis standards of care as they occurred in the simulation model; yellow, contingency standards of care; green, conventional standards of care. The health system had approximately 48 medical intensive care unit (MICU) beds without expansion prior to the pandemic. The New York Ventilator Allocation Guidelines (NYVAG) simulation represents the total number of intubated patients on any day.

Our cohort included 1671 intubated patients at a single hospital system during the NYC spring 2020 surge (Table 1). The crisis period included 674 patients (mean [SD] age 63.7 [13.8] years; 465 male [69.9%]), started on March 31, 2020, and lasted a mean (SD) 15.0 (0.5) days (Figure 2). A total of 571 (84.7%) of all patients ventilated during the crisis period tested positive for COVID-19, 77.5% of individuals within a week of being intubated (eTable 2 in Supplement 1).

**Table 1.** Characteristics of Intubated Patients at a Single Hospital System During the New York City Crisis Period

Characteristic	No. (%) (N = 674)
Age, mean (SD), y	63.7 (13.8)
Sex	
Male	465 (69.9)
Female	209 (31.0)
Race	
African American	82 (12.2)
Asian	67 (9.9)
Native American	4 (0.6)
White	304 (45.1)
Unknown	217 (32.2)
Ethnicity	
Hispanic	173 (25.7)
Non-Hispanic	451 (66.9)
Unknown	50 (7.4)
Past medical history	
Anything	514 (76.1)
Dementia	19 (2.8)
Alzheimer	9 (1.3)
Stroke	59 (8.7)
ALS	0
Parkinson disease	5 (0.6)
Multiple sclerosis	2 (0.3)
Huntington disease	0
Quadriplegic	5 (0.74)
Coma	12 (1.8)
COPD	58 (8.6)
Asthma	62 (9.2)
COPD or asthma	102 (15.1)
Pulmonary fibrosis	6 (0.9)
Pulmonary hypertension	11 (1.6)
Heart failure	56 (8.3)
Coronary artery disease	95 (14.1)
Hypertension	364 (53.9)
Hyperlipidemia	262 (38.8)
Diabetes (type 2)	233 (34.5)
Obesity	127 (18.8)
BMI, mean (SD)	28.83 (8.75)
Q1, Q3	24.3, 33.2
Chronic kidney disease	81 (12.0)
CKD-5/dialysis	34 (5.0)
Cirrhosis	10 (1.48)
Malignant neoplasm	118 (17.5)
Stage IV	4 (3.4)
Gastrointestinal	18 (15.3)
Pulmonary	10 (8.5)
Breast	13 (11.0)
Hematopoietic	8 (6.8)
Current pregnancy	5 (0.7)
Therapies	
Length of mechanical ventilation, Q1, Q2, Q3, d	5.0, 12.0, 25.0
ECMO	23 (3.4)
Surgical procedure (any)	101 (15.0)
Antibiotics	622 (92.1)
Lopinavir/ritonavir	69 (10.2)
Azithromycin	496 (74.5)
Plaquenil	565 (83.7)
Steroids	373 (55.3)
Remdesivir	17 (1.77)
Tocilizumab	199 (29.5)
Continuous paralysis	
Any point during surge period	337 (49.9)
Only within crisis period	282 (41.8)
Survival to discharge	279 (40.6)

Abbreviations: ALS, amyotrophic lateral sclerosis; BMI, body mass index (calculated as weight in kilograms divided by height in meters squared); COPD, chronic obstructive pulmonary disease; ECMO, extracorporeal membrane oxygenation.

Over the 15-day crisis period, a mean (SD) 163.9 (6.1) individuals (95% CI, 151.9-175.8) had their ventilator rationed in our simulations (24.3% of the entire crisis cohort). Of these individuals experiencing ventilator rationing, a mean (SD) of 44.4% (2.8%) (95% CI, 38.3%-50.0%) survived if maintained on a ventilator (observed); whereas only a mean (SD) of 34.8% (2.9%) (95% CI, 28.5%-40.0%) of those individuals receiving a reallocated ventilator survived to discharge (*P* = .06).

Projected survival rates between patients’ front-end triaged priority (step 2) were only significantly different between red (98 of 244 [40.2%]) and blue (11 of 49 [22.5%]) (*P* = .02) but not red and yellow (50 of 161 [31.1%]) or yellow and blue (Table 2). Front-end triage only appeared to exacerbate social disparities for those triaged as yellow and of White race who were more likely to survive than those classified as an unknown race (Table 2; eTable 3 in Supplement 1).

**Table 2.** Survival and Demographic Information for Patients Triaged on the Day of Intubation During the Crisis Period, Selected for Ventilator Rationing, and Who Received a Reallocated Ventilator

Characteristic	Exclusions (step 1)	Front-end triage (step 2)				Back-end triage (step 3)	Total, ventilators rationed, estimated No. (%)^a^	Patients receiving reallocated ventilator, estimated No. (%)^b^
	Patients excluded, No. (%)^c^	Patients classified red, No. (%)	Patients classified yellow, No. (%)	Patients classified blue, No. (%)	Ventilator withheld, estimated No. (%)	Ventilators withdrawn, estimated No. (%)		
Total	2 (100)	244 (100)	161 (100)	49 (100)	9.6 (5.8)	154.3 (94.2)	163.8 (100)	154.3 (100)
Survive, [95% CI]^d^	0	98 (40.2)	50 (31.1)	11 (22.5)	2.21 (23.1) [0-47.1]	70.6 (45.8) [39.9-51.7]	72.8 (44.4) [38.8-50.0]	52.8 (34.4) [28.5-40.0]
SOFA [95% CI]	14.0	4.8 [0.6-9.0]^e^	9.6 [7.4-11.8]^e^	13.3 [12.1-16.5]^e^	NR^f^	NR^f^	NR^f^	NR
Mean age [95% CI], y	NR^g^	63.4 [35.2-91.6]	65.1 [38.5-91.7]	64.2 [40.6-87.8]	63.8 [46.5-81.1]	63.8 [46.5-81.1]	62.7 [36.3-89.1]	64.1 [36.9-91.3]
Male	1 (50.0)	155 (63.5)	118 (73.2)	31 (63.2)	5.39 (43.6)	114 (74.0)	119.7 (73.0)	104.2 (67.5)
Female	1 (50.0)	89 (36.5)	57 (39)	18 (36.7)	4.17 (56.4)	40 (26.0)	44.2 (27.0)	50.1 (32.5)
Race								
African American	NR^g^	26 (10.6)	24 (14.9)	8 (16.3)	1.8 (18.7)	17.3 (11.1)	19.1 (11.6)	18.8 (12.2)
Asian	NR^g^	24 (9.8)	22 (13.6)	5 (10.2)	1.2 (11.9)	14 (9.0)	15.2 (9.2)	18.8 (12.2)
Native American	NR^g^	0	1 (0.6)	1 (2.0)	0.9 (0.5)	0.9 (0.5)	0.9 (0.5)	0.6 (0.4)
Unknown	NR^g^	89 (36.4)	51 (31.6)	16 (32.7)	3.1 (32.7)	46.8 (30.3)	49.9 (30.4)	52.7 (34.1)
White	NR^g^	105 (43.0)	63 (39.1)	19 (38.7)	3.5 (36.2)	75.3 (48.7)	78.8 (48.0)	63.3 (41.0)
Ethnicity								
Hispanic	NR^g^	68 (27.9)	40 (24.8)	16 (32.6)	2.59 (27.1)	34.3 (22.2)	36.7 (22.3)	40.1 (25.9)
Non-Hispanic	NR^g^	156 (63.9)	110 (68.3)	30 (61.2)	5.49 (57.4)	108.2 (70.1)	114.7 (69.9)	102.9 (66.6)
Unknown	NR^g^	20 (8.1)	11 (6.8)	3 (6.1)	1.47 (15.3)	11.8 (7.6)	12.5 (7.6)	11.3 (7.3)

Abbreviations: NR, not reported; NYVAG, New York State Ventilator Allocation Guidelines; SOFA, Sequential Organ Failure Assessment.

^a^ Rationed ventilators include those withheld during step 2 and those withdrawn during step 3.

^b^ Of those newly intubated during the crisis period who received a reallocated ventilator, an estimated 88.6 individuals (57.4%) were red, 53.7 (34.8%) were yellow, and 12.0 (7.8%) were blue. Patients triaged to receive a ventilator were provided any available ventilator at random. Individuals whose ventilator was withheld did not technically have a ventilator to provide to another individual, therefore only ventilators withdrawn could be provided to patients triaged to receive a ventilator.

^c^ One patient was excluded based on exclusionary criteria leaving out patients who experienced, “Cardiac arrest: unwitnessed arrest, recurrent arrest without hemodynamic stability, arrest unresponsive to standard interventions and measures; Trauma-related arrest”; the other experienced, “Traumatic brain injury with no motor response to painful stimulus (ie, best motor response = 1).” One individual was intubated prior to the crisis period and would have their ventilator withdrawn, while the other would have been excluded from receiving a ventilator during crisis period.

^d^ For surviving patients, *P* values for comparisons were as follows: red vs yellow groups, *P* = .06; red vs blue, *P* = .02; yellow vs blue, *P* = .25; total ventilator rationed vs red, *P* = .39; total ventilator rationed vs yellow, *P* = .01; total ventilator rationed vs blue, *P* = .006; total ventilator vs received ventilator, *P* = .06; rationed ventilator withheld vs rationed ventilator withdrawn, *P* < .001.

^e^ For all comparisons, *P* < .001.

^f^ Information available in eFigure 2 in Supplement 1.

^g^ One patient who met NYVAG exclusionary criteria did not have demographic information.

**Figure 3.**
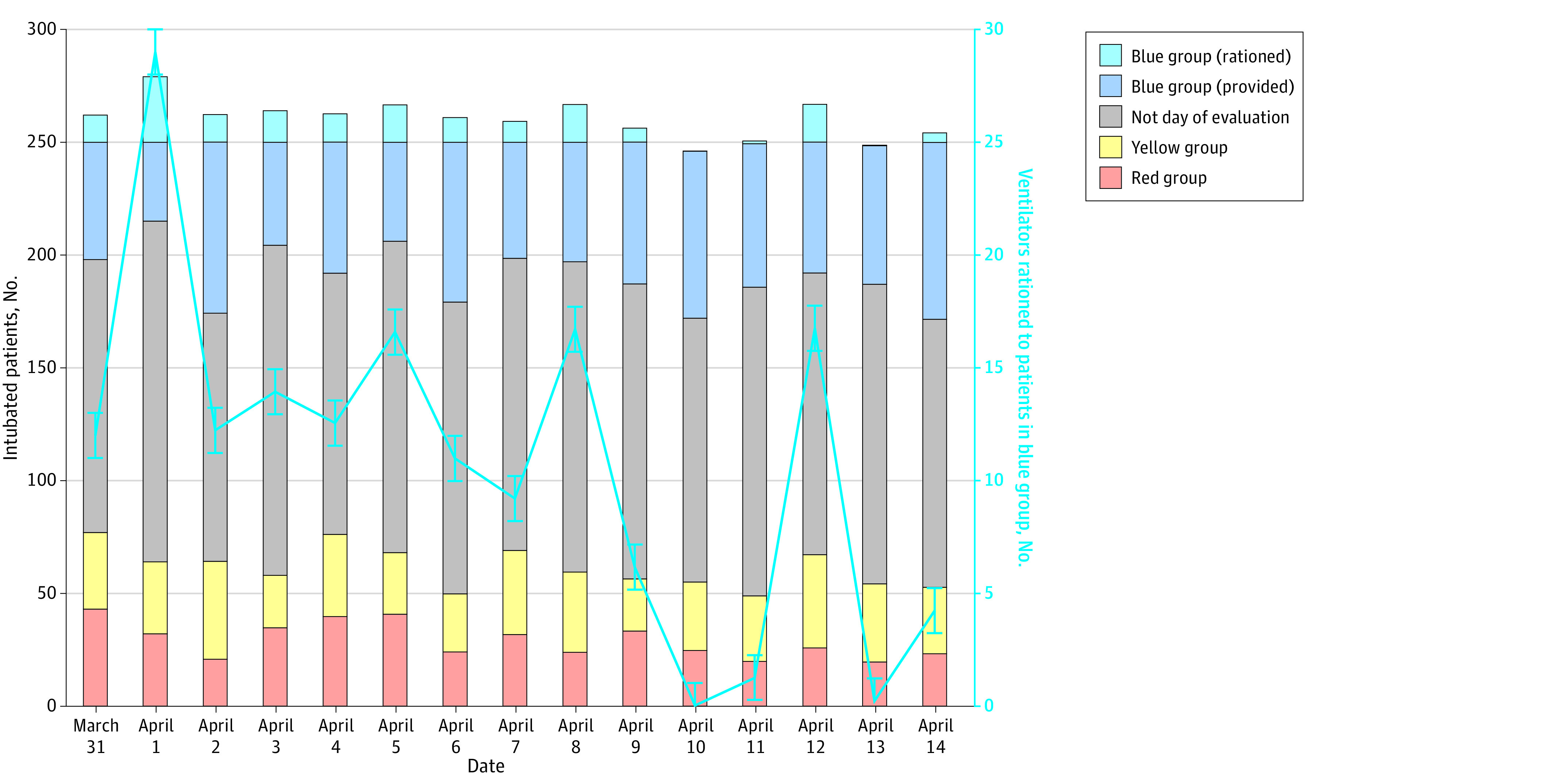
Triage and Rationing of Intubated Patients by NYVAG During the Crisis Period

Ventilators were only rationed from those triaged as blue with a maximal of 29 individuals on a single day (Figure 3; eTable 4 in Supplement 1). Ventilator rationing had 3 subsequent peaks of 16 individuals, and 2 days where less than 1 individual needed their ventilator to be rationed. Those triaged as blue composed 23.9% (59.6 patients) to 35.2% (88.0 patients) of the daily ventilator occupancy and 0% to 45.3% (29.0 patients) of individuals triaged as blue were required to have their ventilator rationed on any day during the crisis period. Of the patients classified as blue and selected for ventilator rationing, 94.8% occurred in those undergoing a ventilator time trial (eFigure 2 in Supplement 1); 45.5% of the patients selected to have their ventilator rationed had been intubated for 7 or more days and had either a SOFA score between 8 and 11 or a SOFA score less than 7 but were not improving compared with the previous SOFA assessment. These patients, combined, survived to hospital discharge 60.6% of the time.

A subsequent simulation model subcategorizing blue by SOFA score on the day of evaluation (iNYVAG) was found to differentiate between estimated survival (Figure 1; eFigure 3 in Supplement 1). Moreover, those patients simulated to have their ventilator rationed were less likely to survive to discharge in iNYVAG (25.3%), compared with NYVAG (44.4%; *P* < .001) (eTable 5 in Supplement 1). iNYVAG did require 23.8 more individuals to have their ventilator rationed (mean [SD], 187.6 [4.2] individuals; 95% CI, 179.5-195.8) over the same duration of the crisis period. eFigure 4 in Supplement 1 displays the criteria frequency for individuals classified as blue and chosen for ventilator rationing under iNYVAG vs NYVAG. iNYVAG did not worsen health disparities (eTable 5 in Supplement 1).

### Missing Data

During the 15-day crisis 674 patients required ventilators, corresponding to 2359 observed patient SOFA days that could be assessed by NYVAG. Neurologic and hepatic subscores were commonly missing, with about half patient SOFA days missing 1 category (eTable 6 in Supplement 1). Sixteen patients were missing a SOFA score on the day of intubation (eTable 7 in Supplement 1).

## Discussion

To our knowledge, this was the first study simulating a CSC protocol using real pandemic strain with realistic resource limitations, which undertook a granular analysis of its performance for both resource allocation and reallocation. Our simulations suggest that by implementing NYVAG during the NYC spring 2020 surge once 95% of prepandemic ventilator supply had been occupied at our health system, a crisis period would have lasted 15.0 days and required 24.3% of the crisis cohort (163.9 individuals) to have had their ventilator rationed. It remains concerning that NYVAG might ration ventilators away from patients with a high chance of survival (44.4%) toward newly intubated patients with a lower chance of survival (34.8%). As evaluated, this CSC rationing strategy would not achieve the intended goal of saving the most lives and might worsen the crisis.

In our study, there was rapid escalation of intubated patients immediately prior to the crisis period, homogenous pathology of intubated patients, and variable ventilator strain during the crisis period. Only 28.0%, 60.8%, and 77.6% of the ventilator supply were utilized at 14, 7, and 5 days prior to the crisis period, respectively, emphasizing the rapid escalation of intubated patients leading to the crisis period and the importance of proactive disaster plans. Second, 84.7% of individuals tested positive for COVID-19, challenging conventional thought that surges requiring resource rationing may be related to multiple diseases.^26^ Third, we found that ventilator strain stuttered (Figure 3). This highlights the variability and severity of rationing that might occur during a prolonged crisis period. Such variable ventilator strain is more likely due to the number of new intubations, deaths, or extubations rather than related to NYVAG performance.

Focusing on the performance of ventilator front-end triage, NYVAG was only able to distinguish patients based on survival at the time of ventilator allocation between those triaged as red and blue but not red and yellow or yellow and blue. This may be related to small sample size or missing SOFA information. Notably, 30.7% of patients front-end triaged as red were missing 3 or more SOFA subscores and had a 20.0% chance of survival to discharge (comparable with those front-end triaged as blue) (eTable 7 in Supplement 1). Further analysis of front-end triage that excluded individuals missing 3 or more SOFA subscores did achieve further front-end prognostic differentiation between red and yellow (*P* = .002) but not yellow and blue (*P* = .24). Some patients may require front-end triage decisions with marginal information^11^ (eg, patients arriving in extremis, as possibly experienced for 16 patients) or supply chain fluctuations, which may limit laboratory testing.^38^ The limited ability of NYVAG to provide front-end prognostic discrimination, which is worsened by incomplete SOFA information, highlights the importance of back-end triage.

The potential reallocation of ventilators after a time trial, or back-end triage, accounted for 94.8% of all ventilator rationing. This reallocation, as others have suggested,^25^ prompts a paradigm shift in CSC planning to emphasize a time trial. A concerning feature is that NYVAG removed ventilators from 1 patient and provided them to another individual with a lower chance of survival. NYVAG’s poor survival performance may be because NYVAG randomly selected among all individuals with blue classification without further prognostic subcategorization. iNYVAG was found to improve triage efficiency without increasing the length of the crisis period or worsening social disparities (eFigure 5 and eTable 8 in Supplement 1). With respect to those individuals iNYVAG selected for ventilator rationing, several observations can be emphasized. First, the same proportion occurred during back-end triage. Second, the total number of rationed individuals was increased because NYVAG maintained ventilators in those likely to expire, which became an available ventilator upon the patient’s expiration. Third, the same 81 individuals were simulated for ventilator rationing in each simulation (43.2% of all individuals simulated for ventilator rationing within each simulation; eFigure 5 in Supplement 1), and likely related to subcategorization and patient throughput. While such a modification to NYVAG’s triage (iNYVAG) achieves the primary goal of saving the most lives, it may place additional burdens on a triage committee and highlights the importance of palliative care integration.

While we assert that a well-functioning triage committee might be able to identify prognostic nuances a SOFA-based guideline cannot, we remain concerned regarding the high number of individuals that might need to be selected for ventilator reallocation (peaking at 29 out of a total of 60 patients triaged as blue on 1 day, and 163.9 over a 15-day period). Such reliance on a well-functioning multidisciplinary triage committee may place additional staffing burdens on an already overstrained system and may increase subjectivity to the process.

While we remain concerned about the inability of a static SOFA score to predict survival,^11^ we believe that the analysis of SOFA prognostication in CSC simulations remain incomplete because they do not account for back-end triage. One prospective study of 103 consecutive critically ill COVID-19 patients found the change in SOFA scores between the day of admission and 48 hours later was not predictive for ICU survival^39^; however, our study suggests that 72.8% of patients had their ventilator reallocated after 48 hours. While there is growing awareness of SOFA’s challenges in assessing survival for front-end triage, there is a lack of studies evaluating its effectiveness after a trial of critical care. Utilization of an objective prognostication score does have certain advantages. It is objective, promotes consistency, fairness, transparency, may reduce clinician distress or guilt regarding resource rationing, and provides a measure for accountability.^37^

It is encouraging that NYVAG did not appear to worsen health disparities by exclusion, front-end triage, or back-end triage by age, race, or ethnicity. This conclusion must be interpreted with caution as most of our crisis cohort were White or non-Hispanic and these individuals were intubated earlier in the surge compared with those whose race could not be identified (unknown) or Hispanic. While this may be indicative of an underlying health disparity, our study was not designed to analyze the timing of intubation. Future studies should scrutinize outcome disparities among a crisis cohort that evolves throughout the crisis period.

### Strengths and Limitations

We believe our cohort is novel in its patient selection, disease composition, and our conclusions remain generalizable. Our cohort not only includes anyone requiring a ventilator, but also only those requiring a ventilator during the crisis period, which is consistent with how CSC would be practically applied.^35^ Furthermore, our COVID-19 cohort is generalizable to other COVID-19 patients experiencing an extreme surge.^40,41,42,43,44^ The high observed mortality was limited to the crisis period, consistent with other local reports during the study period,^21,28,45,46^ and related to the unprecedented severe ICU strain that would be consistent in communities activating CSC.^46^

This study had several potential limitations. First, related to our simulation model, we adjusted the hour that patients were intubated, extubated, expired, or placed on extracorporeal membrane oxygenation for simplicity, and the documented hour may not be reliable. This precluded iterative lotteries as what would occur throughout the day in a clinical setting, and our model assigned available ventilators at random to patients triaged for intubation (step 2). While this may be partially responsible for the triage inefficiency of NYVAG or iNYVAG, it does not minimize the clinical importance that iNYVAG directs ventilators to those individuals with a higher chance of surviving.

Second, our retrospective study suffered from missing SOFA information and is vulnerable to a missing data bias. The missing neurology subscore, similar to other comparable studies,^19,27^ may be related to practices at the time limiting bedside assessment or that 41.8% of patients required paralytics. Missing laboratory data (such as hepatic function) may be related to the test not being clinically indicated at the time. We believe some missing SOFA data approximates realistic limitations during a pandemic surge, as supply chain limitations related to laboratory testing are anticipated to occur.^38^ It is reassuring that 1 study did not find the hepatic or neurologic SOFA subscores to be play a large role in assessing mortality compared with the pulmonary, circulatory, or renal subscores.^47^ Another simulation rationing study,^27^ besides treating missing SOFA subscores as zero and performing a neurologic subscore sensitivity analysis, also assumed missing SOFA subscores were missing at random and performed a missing imputation analysis. The study concluded that missing data were unlikely to have materially influenced their results. Unfortunately, missing SOFA subscores do not occur at random in our cohort but are exacerbated during the crisis period of the surge, and other proposed solutions^48,49^ would be insufficient or not represent a pragmatic evaluation of NYVAG’s performance.

Our study may therefore introduce missing data bias as we replaced missing subscores with zero, lowering overall patient SOFA scores, and might have made some patients appear healthier than they actually were. This may manifest in 2 ways. First it may impede efficient ventilator rationing because it may “overprioritize” some patients during front-end triage or during back-end triage by patients not otherwise satisfying the moderately high SOFA score that might triage them as blue (eTable 7 in Supplement 1). Second, because patients can also be triaged as blue if they experience a worsening SOFA comparison between current and previous assessments, missing data may either facilitate or hinder ventilator reallocation if missing data are predominantly on the previous or current assessment respectively. We did not find that missing data biased our back-end triage results substantially as missing data are likely to occur in the setting of disaster or clinical circumstances, a reported SOFA subcategory did not provide an accurate estimate of the magnitude of organ dysfunction, and only 47.3% of patients selected for ventilator rationing were because of a worsening SOFA comparison. A disaster plan that requires a complete SOFA score may achieve greater prognostic accuracy between triage priority categories and will minimize the risk of missing data bias.

## Conclusion

This simulated cohort study represents, to our knowledge, the first descriptive performance of a CSC that incorporates real pandemic strain, actual patient throughput, and both front-end and back-end triage. Implementing NYVAG during the NYC spring 2020 surge at 1 institution was simulated to have diverted ventilators toward patients with a lower chance of survival. Further subcategorization of those with the lowest priority for ventilators, such as was instituted with iNYVAG, may help alleviate this concern. Improving CSC should focus on refining back-end triage. It remains reassuring that NYVAG did not appear to worsen social disparities of health.

## Notes

Supplement 1.
eMethods.
**eTable 1.** Phases of NYVAG Simulation **eTable 2.** Timing of COVID-19 Testing With Respect to Intubation **eTable 3.** Survival to Hospital Discharge by Race/Ethnicity on Day of Intubation During the Crisis Period **eTable 4.** Triage and Rationing of Intubated Patients by NYVAG During the Crisis Period **eTable 5.** iNYVAG Survival and Demographic Information for Those Triaged as Blue and Selected for Ventilator Reallocation, and Those Receiving a Reallocated Ventilator **eTable 6.** Missing SOFA Scores, Including Subcategories, for Any Day a Patient Was Being Reassessed by the NYVAG **eTable 7.** Missing SOFA Scores, Including Subcategories, for Patients Triaged on the Day of Consideration for Intubation During the Crisis Period **eTable 8.** Characteristics of Individuals Selected for Ventilator Rationing That Occurred for All iNYVAG Simulations **eFigure 1.** NYVAG Step 1 Exclusionary Criteria **eFigure 2.** Classification of Blue Patients Selected Have Their Ventilator Rationed With Their Observed Survival to Discharge (NYVAG) **eFigure 3.** Survival to Discharge by Blue Subcategorization for NYVAG and iNYVAG **eFigure 4.** Classification of Blue Patients Selected Have Their Ventilator Rationed With Their Observed Survival to Discharge (NYVAG vs iNYVAG) **eFigure 5.** How Often the Same Individual Was Selected for Ventilator Rationing Across All Simulations by Rationing Strategy

Supplement 2.
Data Sharing Statement
