# Supplementary material for: Simulation of New York City’s Ventilator Allocation Guideline During the Spring 2020 COVID-19 Surge
Source: JAMA Netw Open. 2023 Oct 5;6(10):e2336736. doi: 10.1001/jamanetworkopen.2023.36736 (PMC10556967; doi:10.1001/jamanetworkopen.2023.36736)
Supplement: Supplement 1. — eMethods. eTable 1. Phases of NYVAG Simulation eTable 2. Timing of COVID-19 Testing With Respect to Intubation eTable 3. Survival to Hospital Discharge by Race/Ethnicity on Day of Intubation During the Crisis Period eTable 4. Triage and Rationing of Intubated Patients by NYVAG During the Crisis Period eTable 5. iNYVAG Survival and Demographic Information for Those Triaged as Blue and Selected for Ventilator Reallocation, and Those Receiving a Reallocated Ventilator eTable 6. Missing SOFA Scores, Including Subcategories, for Any Day a Patient Was Being Reassessed by the NYVAG eTable 7. Missing SOFA Scores, Including Subcategories, for Patients Triaged on the Day of Consideration for Intubation During the Crisis Period eTable 8. Characteristics of Individuals Selected for Ventilator Rationing That Occurred for All iNYVAG Simulations eFigure 1. NYVAG Step 1 Exclusionary Criteria eFigure 2. Classification of Blue Patients Selected Have Their Ventilator Rationed With Their Observed Survival to Discharge (NYVAG) eFigure 3. Survival to Discharge by Blue Subcategorization for NYVAG and iNYVAG eFigure 4. Classification of Blue Patients Selected Have Their Ventilator Rationed With Their Observed Survival to Discharge (NYVAG vs iNYVAG) eFigure 5. How Often the Same Individual Was Selected for Ventilator Rationing Across All Simulations by Rationing Strategy [file jamanetwopen-e2336736-s001.pdf]

Supplemental Online Content

Walsh BC, Zhu J, Feng Y, et al. Simulation of New York City's ventilator allocation guideline during the spring 2020 COVID-19 surge. *JAMA Netw Open.* 2023;6(10):e2336736.
doi:10.1001/jamanetworkopen.2023.36736

eMethods

eTable 1. Phases of NYVAG Simulation

eTable 2. Timing of COVID-19 Testing With Respect to Intubation

eTable 3. Survival to Hospital Discharge by Race/Ethnicity on Day of Intubation During the Crisis Period

eTable 4. Triage and Rationing of Intubated Patients by NYVAG During the Crisis Period

eTable 5. iNYVAG Survival and Demographic Information for Those Triaged as Blue and Selected for Ventilator Reallocation, and Those Receiving a Reallocated Ventilator

eTable 6. Missing SOFA Scores, Including Subcategories, for Any Day a Patient Was Being Reassessed by the NYVAG

eTable 7. Missing SOFA Scores, Including Subcategories, for Patients Triaged on the Day of Consideration for Intubation During the Crisis Period

eTable 8. Characteristics of Individuals Selected for Ventilator Rationing That Occurred for All iNYVAG Simulations

eFigure 1. NYVAG Step 1 Exclusionary Criteria

eFigure 2. Classification of Blue Patients Selected Have Their Ventilator Rationed With Their Observed Survival to Discharge (NYVAG)

eFigure 3. Survival to Discharge by Blue Subcategorization for NYVAG and iNYVAG

eFigure 4. Classification of Blue Patients Selected Have Their Ventilator Rationed With Their Observed Survival to Discharge (NYVAG vs iNYVAG)

eFigure 5. How Often the Same Individual Was Selected for Ventilator Rationing Across All Simulations by Rationing Strategy

© 2023 Walsh BC et al. *JAMA Network Open.*

This supplemental material has been provided by the authors to give readers additional information about their work.

© 2023 Walsh BC et al. *JAMA Network Open.*

Supplemental Methods:
Ventilator use in the real world was queried from the health record however because of incomplete ventilator documentation, ventilator use was amended in the following manner to represent real continuous use. Ventilator use was amended if two continuous days were missing but three continuous days before and after were documented (90), two continuous days before and one day after were documented (32), or one day before and after (12); one missing day but three continuous days before and after were documented (167), two continuous days before and after were documented (52), or one day before and after were documented (169). Amending in this manner accounted for 2.1% (513/24,138) of all patient-ventilator-days during the surge. Patients with subsequent ventilator use were otherwise considered to be reintubated and were considered for ventilator allocation (Step 2). Because extubation was not documented reliably, the time-step of our simulation was daily.

The time of real patient events (intubation, extubation, & death) but not the day were modified for the simulation model. SOFA scores were obtained for each mechanically ventilated patient daily as close to 10:00am as possible. Missing pulmonary SOFA subscores used pulmonary mSOFA if available.[1, 2] However, if pulmonary mSOFA or any other SOFA subscore was missing it was presumed normal (0), as has been practiced in similar studies and pragmatic in that is likely what actually occur.[1, 3, 4, 5] If there was no reported SOFA score on the day of consideration for intubation, the SOFA score from the next day was used instead for the day of consideration for intubation and any relevant comparisons.[3] NYVAG triage, including patient intubation if assigned, was simulated to occur at 10:00am. Patients who had their ventilator rationed were simulated to expire.[6, 7] Patients who were extubated, died, or placed on extracorporeal membrane oxygenation (ECMO) were simulated to occur at 23:00pm. Patients exited the simulation when placed onto ECMO[3] because (1) NYVAG did not include ECMO, (2) the pulmonary SOFA subscore might not be representative of the severity of respiratory illness for patients on ECMO, and (3) institutional extubation practices vary while patients are on ECMO.

An initial premises of this study was to determine what may have occurred if NYVAG was implemented without additional ventilators. To that end, we defined the initiation of our crisis period as once 95% of our pre-pandemic supply of ventilators became occupied. Retrospectively, this supply corresponds to 71.8% of the observed peak ventilator use and is of comparable strain to what others have simulated.[8]

The following is an expanded description of the model used to simulate NYVAG (eTable 1). All patients who were intubated in the real world during the crisis period would undergo NYVAG Step 1 and be excluded from receiving or maintaining a simulated ventilator if exclusionary criteria 1, 3, or 4 were satisfied by admission diagnosis and chart review (performed and evaluated by BCW, concurred by DP). Criteria 2 & 5 could not be objectively or reliably satisfied.

Those patients not excluded by Step 1 and under consideration for the initiation of mechanical ventilation proceeded to Step 2 or the potential allocation of ventilators. Step 2 is facilitated by calculating that patient’s SOFA score on the day of consideration. Those patients already

intubated, and thus possessing a ventilator, prior to the start of the crisis period would proceed to Step 3 or the potential reallocation of ventilators. The length of time an individual possessed a ventilator, including time prior to the crisis period, would be used to determine which NYVAG reassessment day would be applied (ie. A patient that was ventilated for 4 days prior to the start of the crisis period, would use the Day 5 NYVAG reassessment criteria on the day starting the crisis period)[9, pg 14, 53, 61]. Patients were triaged to Red (highest priority to receive a ventilator), Yellow (intermediate priority), or Blue (ineligible for a ventilator unless a surplus of ventilators exists) by Step 2 or Step 3. After the triage of patients, patients were next designated if they were to receive or maintain a ventilator. This designation process occurred in the following sequence: first, patients undergoing their time trial (between NYVAG reassessment days) were randomly selected; then, patients triaged as Red were chosen at random; followed by patients triaged as Yellow at random, and finally those triaged as Blue at random. Patients were designated to receive or maintain a ventilator in this manner until the ventilator supply (250) was exhausted.

As the model allowed patients within the same triage category to be designated to receive a ventilator at random until ventilator supply was exhausted, the specific individual who received a ventilator may vary for each simulation. For example, if on the first day of the crisis period 64 individuals were triaged as Blue but there were only 52 ventilators remaining, each simulation would require 12 Blue individuals, selected at random, to experience ventilator rationing (and simulated to expire). Depending on which 12 are randomly selected, the subsequent days rationing needs may differ. An individual who was selected to maintain their ventilator in one simulation but expired after NYVAG ventilator assignment will have their ventilator return to supply the subsequent day as an available-ventilator (decreasing the need to ration a ventilator by 1). If the same individual in another simulation was otherwise selected for ventilator reallocation, that ventilator will be provided to another individual and (as long as that patient does not expire that day) will not be an available-ventilator the subsequent day. Important outcomes of one simulation of the crisis period, such as the number of individuals experiencing ventilator rationing or the length of the crisis period, may differ from another based which individuals are selected for ventilator rationing. To account for this random selection, 10,000 simulations of the crisis period were performed. To define the crisis cohort used in the analysis, we included 95% of all simulations with the shortest crisis period.

Patients identified to maintain a ventilator continued their previously assigned ventilator. The following was used to identify newly intubated patients who received a reallocated ventilator (a subset of Step 2 and a focus of back-end triage) vs those who received an available-ventilator. At 23:00pm for each day of the crisis period, the total ventilators from those patients who were extubated, expired, or placed on ECMO would count as available-ventilators for the following day. The number of available-ventilators would be randomly provided to newly intubated patients identified to receive a ventilator per NYVAG (Step 2). Any additional newly intubated patients designated to receive a ventilator obtained a ventilator at random from those patients identified to have their ventilator reallocated (Step 3). NYVAG identified which patients were prioritized and to receive a ventilator, however ventilators were assigned at random to those individuals whom NYVAG identified to receive a ventilator.

Our simulation model allowed for newly intubated patients to potentially receive a ventilator from a patient of the same triage category. NYVAG explicitly state that patients cannot be compared to one another when they are within the same triage category (i.e., new vs old).[9, pg 60, 68] The text is inconsistent in specifying how supply shortages within a triage category should be addressed; in one place it suggests either a first-come first-served or lottery may be acceptable secondary allocation strategy[9, pg 60], while in another it only mentions a lottery [9, pg 68]. While footnote 139 challenges our interpretation, we utilized a lottery as a secondary allocation strategy because the language clearly cautions against a first-come first-served allocation strategy [9, pg 42, 61, 68] and our institution would have followed this interpretation of the model.

Our analysis focuses on front-end triage (Step 2, those triaged as Red, Yellow, or Blue on the day considered for intubation) and back-end triage (Step 3, those who had their ventilator reallocated) because individuals undergoing a Step 3 time trial evaluation can be triaged to different priorities throughout the crisis period while maintaining a ventilator and different patients may ultimately be selected for ventilator rationing. Individuals experiencing front-end triage do not vary from simulation to simulation, whereas those selected to receive a ventilator or have their ventilator reallocated do.

An amendment to NYVAG model (iNYVAG, Figure 1) whereby the Blue category was subcategorized to incorporate the acuity of illness on the day of evaluation is described as follows. Ventilators would be first rationed by subcategory as follows: Blue individuals whose SOFA score was > 11 after already receiving a time trial ($Blue_1$), then from Blue individuals considered for intubation but with a SOFA > 11 or those with a SOFA score between 8-11 after a time trial ($Blue_2$), and finally from those Blue individuals with a SOFA < 7 after a time trial ($Blue_3$).

Race and ethnicity are self-reported by the patient or identified unknown if unable or unwilling to self-identify. Categorical data were analyzed with the chi-square test using the 95% confidence interval (CI). Continuous variables were compared with a two-tailed t-test using 5% significance level.

References:

1. Wunsch H, Hill AD, Bosch N, et al. Comparison of 2 Triage Scoring Guidelines for Allocation of Mechanical Ventilators. *JAMA Netw Open*. Dec 1 2020;3(12):e2029250. doi:10.1001/jamanetworkopen.2020.29250
2. Knaus WA, Wagner DP, Draper EA, et al. The APACHE III prognostic system. Risk prediction of hospital mortality for critically ill hospitalized adults. *Chest*. Dec 1991;100(6):1619-36. doi:10.1378/chest.100.6.1619
3. Walsh BC, Pradhan D, Mukherjee V, Uppal A, Nunnally ME, Berkowitz KA. How Common SOFA and Ventilator Time Trial Criteria would have Performed during the COVID-19 Pandemic: An Observational Simulated Cohort Study. *Disaster Med Public Health Prep*. Jun 9 2022:1-25. doi:10.1017/dmp.2022.154

© 2023 Walsh BC et al. *JAMA Network Open.*

4. Jezmir JL, Bharadwaj M, Chaitoff A, et al. Performance of crisis standards of care guidelines in a cohort of critically ill COVID-19 patients in the United States. *Cell Rep Med*. 2021;2(9):100376-100376. doi:10.1016/j.xcrm.2021.100376
5. Molina MF, Cash RE, Carreras-Tartak J, et al. Applying crisis standards of care to critically ill patients during the COVID-19 pandemic: Does race/ethnicity affect triage scoring? *J Am Coll Emerg Physicians Open*. Aug 2021;2(4):e12502. doi:10.1002/emp2.12502
6. Bhavani SV, Luo Y, Miller WD, et al. Simulation of Ventilator Allocation in Critically Ill Patients with COVID-19. *Am J Respir Crit Care Med*. Sep 9 2021;doi:10.1164/rccm.202106-1453LE
7. Kanter RK. Would triage predictors perform better than first-come, first-served in pandemic ventilator allocation? *Chest*. Jan 2015;147(1):102-108. doi:10.1378/chest.14-0564
8. Chuang E, Grand-Clement J, Chen JT, Chan CW, Goyal V, Gong MN. Quantifying Utilitarian Outcomes to Inform Triage Ethics: Simulated Performance of a Ventilator Triage Protocol under Sars-CoV-2 Pandemic Surge Conditions. *AJOB Empir Bioeth*. Jul-Sep 2022;13(3):196-204. doi:10.1080/23294515.2022.2063999
9. New York State Task Force on Life and the Law. Ventilator Allocation Guidelines. 2015. https://www.health.ny.gov/regulations/task_force/reports_publications/docs/ventilator_guidelines.pdf. Accessed 4/6/2020.

© 2023 Walsh BC et al. *JAMA Network Open.*

eTable 1: Phases of NYVAG Simulation

KEY:

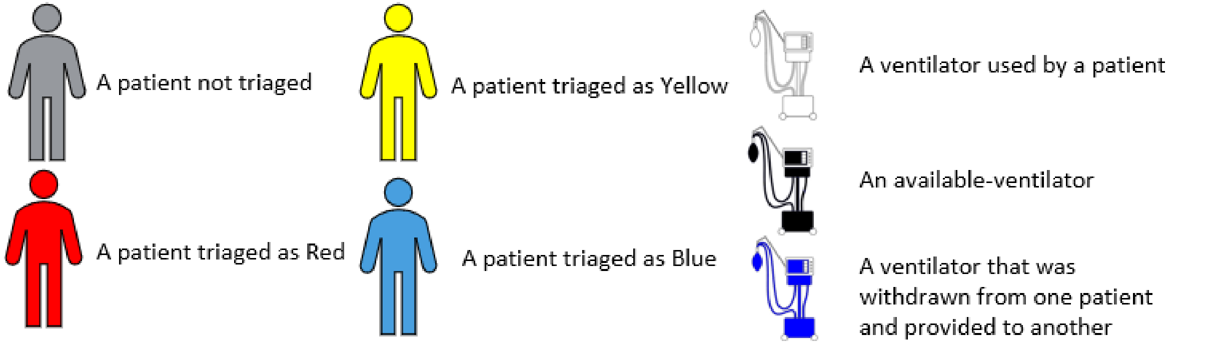

- Numbers on a patient describe the length that patient has been mechanically ventilated (days)
- Each simulation is of the entire crisis period. It repeats phases 1-13 for each day of the crisis period until ventilator demand is less than or equal to 250. Because randomization is used in phases 5 and 8, each simulation of the crisis period

**Phase**	**Day**	**Diagram**	**Description:**
1	0	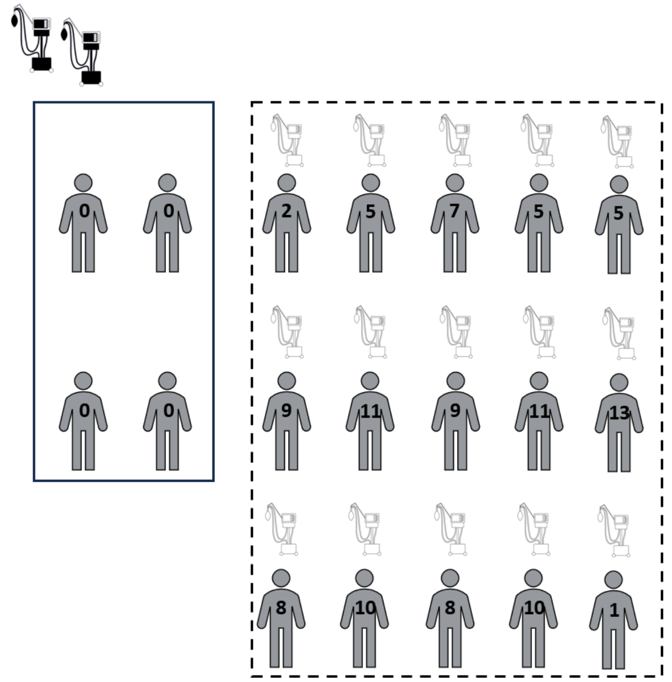	- An example of the NYVAG phases using an initial start of 15 previously ventilated patients (dashed box), each with one of 15 assigned ventilators, 4 patients being considered for intubation (solid box), and 2 available-ventilators. In this example there are 17 total ventilators available for patient use.<br>- Any patient being considered for intubation (solid box) or already possessing a ventilator (dashed line) at the start of the crisis period undergoes NYVAG Step 1.

© 2023 Walsh BC et al. *JAMA Network Open.*

2	0	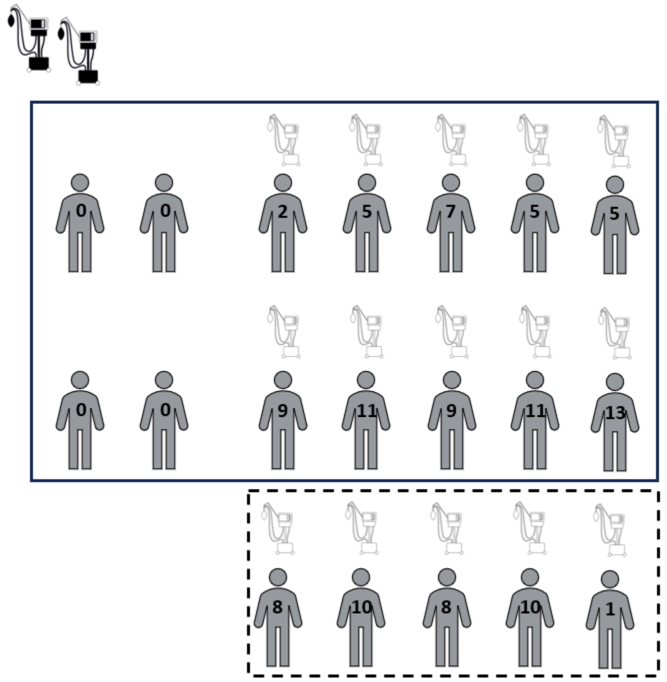	- The solid box describes patients who are being evaluated or re-evaluated for ventilator distribution.<br>- The dashed box describes patients undergoing a time trial and between NYVAG reassessment days.
3	0	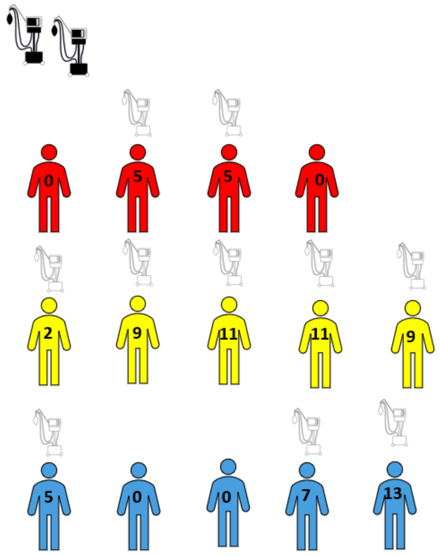	- Patients undergo triage categorization into ventilator priority. Red being prioritized first, followed by Yellow, followed by Blue.<br>- Those being considered for intubation undergo Step 2, while those previously assigned a ventilator undergo Step 3.<br>- Triage categorization is now complete however ventilators have not yet been assigned.

© 2023 Walsh BC et al. *JAMA Network Open.*

4	0	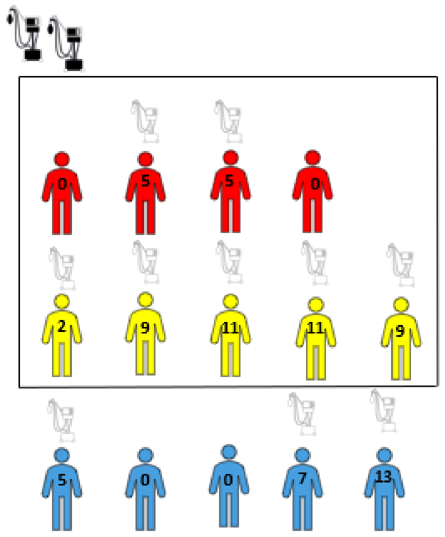	- Ventilators are first provided at random to all patients triaged as Red, then at random to all patients triaged as Yellow, and then at random to all patients triaged as Blue.<br>- In this example, all patients triaged as Red and Yellow are assigned a ventilator (box) however we have not yet determined if these individuals will receive an available-ventilator or a to-be rationed ventilator. This is determined in phases 5-9.<br>- Amongst all of the patients triaged as Blue, only 3 will receive a ventilator (decided by lottery).
5	0	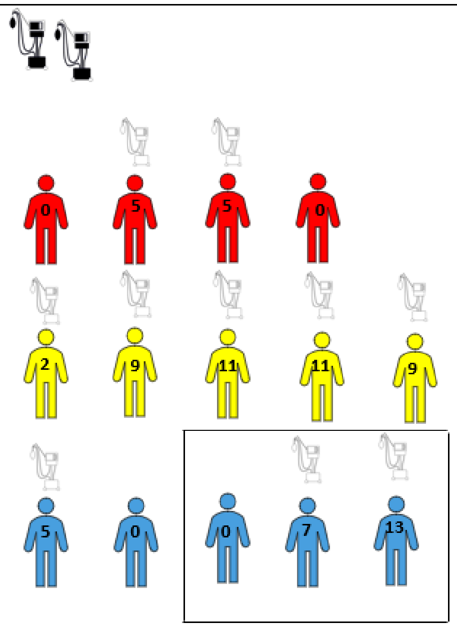	- These are the 3 (out of 5 individuals triaged as Blue) randomly selected to receive or maintain a ventilator. Of these, 2 will be continued on a ventilator, and 1 will be newly receiving a ventilator.

© 2023 Walsh BC et al. *JAMA Network Open.*

6	0	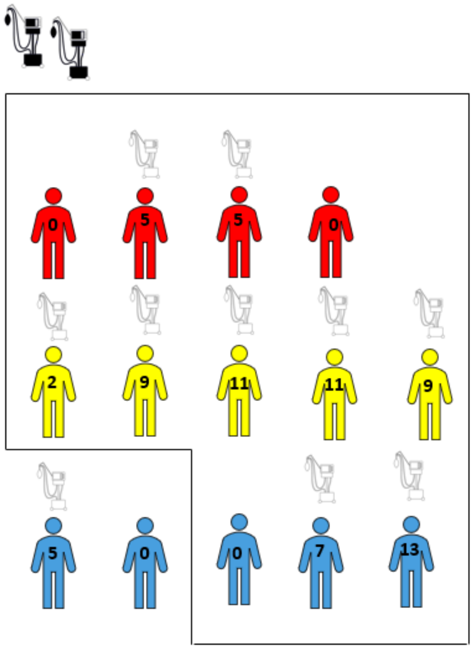 	- At this point, we have now determined which 13 / 15 individuals will receive a ventilator.
7	0	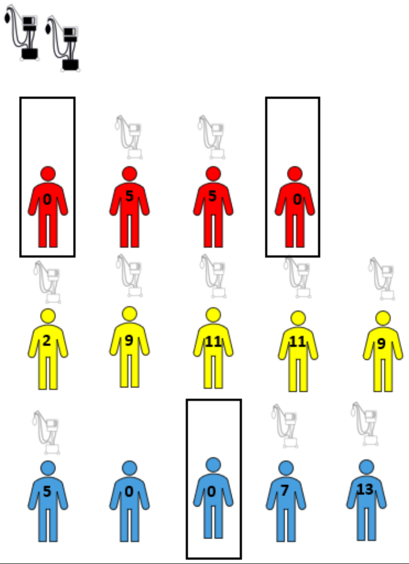 	- Phases 8-10 assign ventilators to those new individuals identified to receive a ventilator.<br>- These 3 individuals in the solid boxes represent new individuals identified to receive a ventilator.
8	0	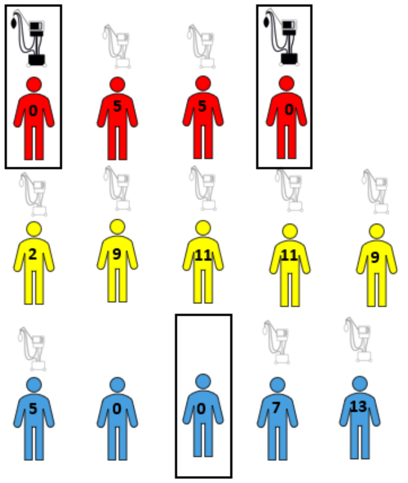 	- In this phase, the two available-ventilators are randomly assigned to the 2 patients triaged as Red.

© 2023 Walsh BC et al. *JAMA Network Open.*

9	0	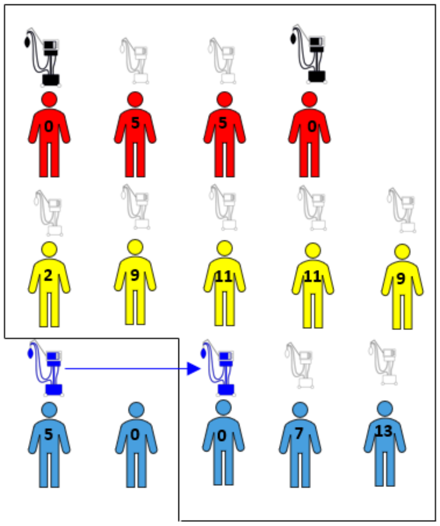	- The black box identifies all patients to be assigned a ventilator by the NYVAG triage process (Phase 7).<br>- The remaining patient triaged as blue and identified to receive a ventilator will receive their ventilator (colored blue) from another Blue patient not selected to continue ventilation.
10	0	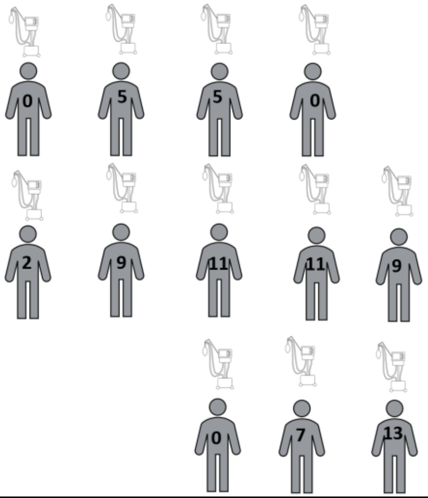	- All patients assigned a ventilator for day 0 by NYVAG.
11	0	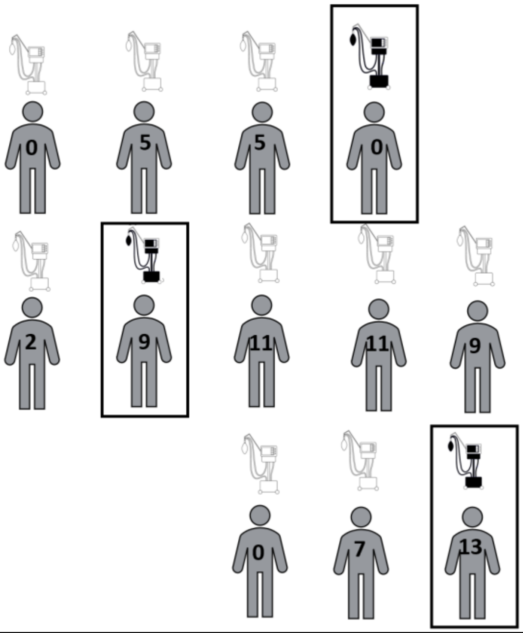	- Patients that expire, are extubated, or placed on ECMO no longer require a ventilator. This is simulated to occur at the end of the day after ventilators are assigned. These three ventilators become available-ventilators for the subsequent day.

© 2023 Walsh BC et al. *JAMA Network Open.*

12	0	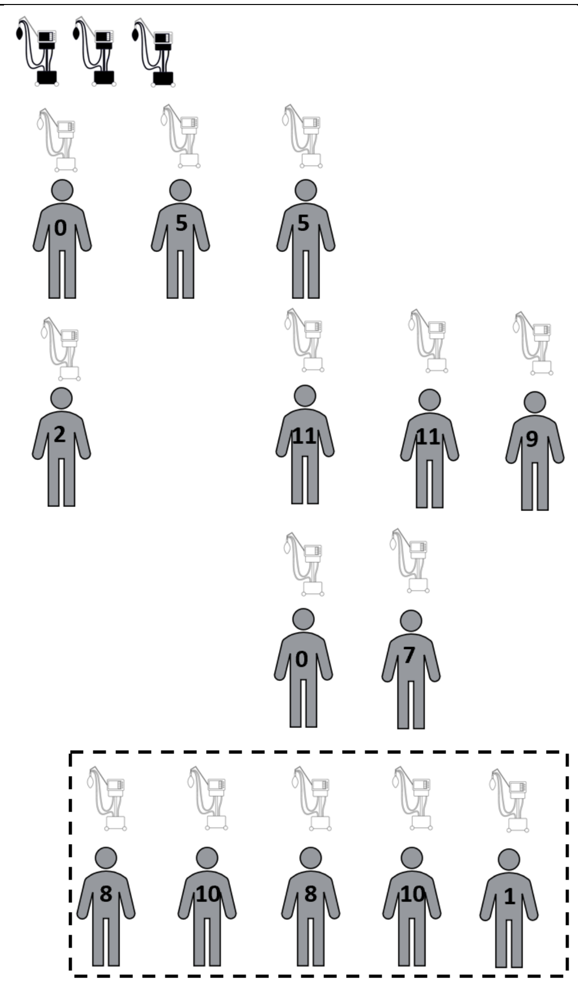	- End of day 0.<br>- The dashed box describes the 5 patients undergoing a time trial and between NYVAG reassessment days (from Phase 2)

© 2023 Walsh BC et al. *JAMA Network Open.*

13	1	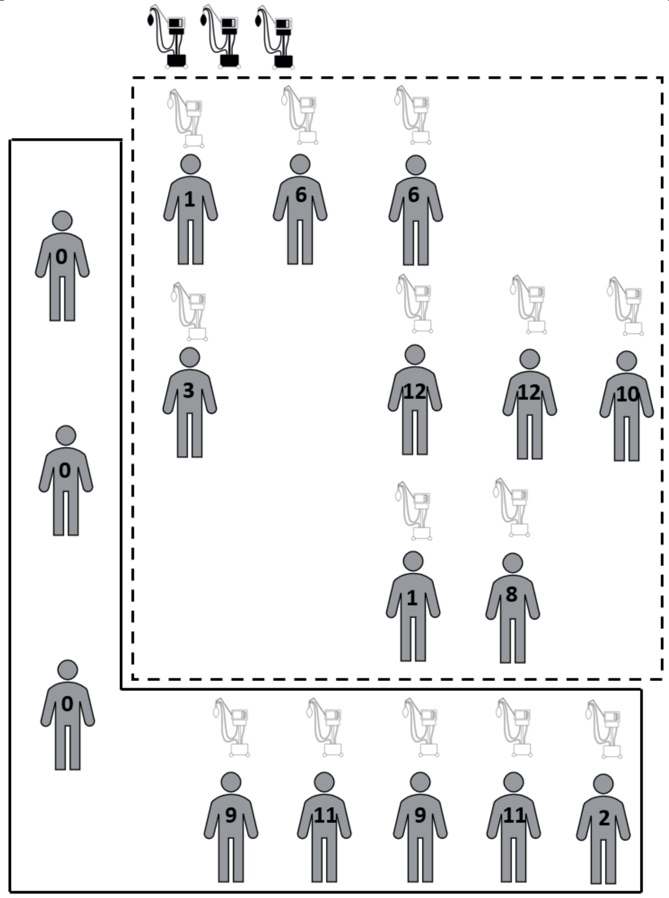	- Day 1<br>- Three new patients are being considered for intubation. These patients would undergo NYVAG Step 1 (Phase 1).<br>- The black solid box includes all patients that would be evaluated by NYVAG for receiving or maintaining a ventilator.<br>- The dashed line includes all patients that are undergoing a ventilator time trial and not due for NYVAG reassessment.<br>- The next phase would be similar to Phase 3.<br>- *The astute observer will note that all patients on this day will be able to receive a ventilator. This will occur whenever the number of patients being considered for intubation is less than or equal to the number of available-ventilators.*

© 2023 Walsh BC et al. *JAMA Network Open.*

eTable 2: Timing of COVID-19 Testing with respect to Intubation

	**Pre-Intubation**			**Post-Intubation**				**Post-Extubation**	**Post-Discharge**
**Time (weeks)**	**> 2**	**1-2**	**0-1**	**0-1**	**1-2**	**2-4**	**4-8**		
**Positive n (%)**	5 (0.74)	46 (6.81)	350 (51.9)	172 (25.5)	1 (0.14)	0 (0)	3 (0.4)	9 (1.3)	0 (0)
**Total Positive n (%)**	571 (84.7)								

© 2023 Walsh BC et al. *JAMA Network Open.*

eTable 3: Survival to Hospital Discharge by Race / Ethnicity on Day of Intubation during the Crisis Period

NYVAG	Newly Intubated Red			Newly Intubated Yellow			Newly Intubated Blue		
	Survived n (%)	Deceased n (%)	p	Survived n (%)	Deceased n (%)	p	Survived n (%)	Deceased n (%)	p
Total	98 (100)	146 (100)	-	50 (100)	111 (100)	-	11 (100)	38 (100)	-
Male	66 (67.3)	89 (60.9)	0.31	34 (68.0)	84 (75.6)	0.31	8 (72.7)	23 (60.5)	0.46
Female	32 (32.7)	57 (39.0)	0.31	16 (32.0)	27 (24.3)	0.31	3 (27.3)	15 (39.5)	0.46
Race									
African American	12 (12.2)	14 (9.5)	0.51	9 (18.0)	15 (13.5)	0.46	1 (9.0)	7 (18.4)	0.46
Asian	7 (7.1)	17 (11.6)	0.25	5 (10)	17 (15.3)	0.36	0 (0)	5 (13.1)	-
Native American	0 (0)	0 (0)	-	0 (0)	1 (0.9)	-	0 (0)	1 (2.6)	-
Unknown	33 (33.6)	56 (38.3)	0.46	10 (20.0)	41 (36.9)	**0.03**	4 (36.3)	12 (31.5)	0.77
White	46 (46.9)	59 (40.4)	0.31	26 (52.0)	37 (33.3)	**0.03**	6 (54.5)	13 (34.2)	0.22
Ethnicity									
Hispanic	24 (24.4)	44 (30.1)	0.34	8 (16.3)	32 (28.5)	0.08	5 (45.4)	11 (28.9)	0.30
Non-Hispanic	69 (70.4)	87 (59.5)	0.09	39 (77.5)	71 (64.2)	0.08	6 (54.5)	24 (63.1)	0.61
Unknown	5 (5.1)	15 (10.2)	0.15	3 (6.1)	8 (7.1)	0.78	0 (0)	3 (7.8)	-

© 2023 Walsh BC et al. *JAMA Network Open.*

eTable 4: Triage & Rationing of Intubated Patients by NYVAG during the Crisis Period

NYVAG	3/31 n (%)	4/1 n (%)	4/2 n (%)	4/3 n (%)	4/4 n (%)	4/5 n (%)	4/6 n (%)	4/7 n (%)	4/8 n (%)	4/9 n (%)	4/10 n (%)	4/11 n (%)	4/12 n (%)	4/13 n (%)	4/14 n (%)	Total
Not Day of Evaluation	121.0 (48.4)	151.0 (60.4)	110.0 (44)	146.3 (58.5)	115.8 (46.3)	138.1 (55.2)	129.4 (51.8)	129.5 (51.8)	137.6 (55)	130.8 (52.3)	117.0 (47.6)	136.9 (54.9)	124.9 (50)	132.8 (53.4)	118.8 (47.5)	
Red	43.0 (17.2)	32.0 (12.8)	20.8 (8.3)	34.7 (13.9)	39.7 (15.9)	40.8 (16.3)	24.0 (9.6)	31.7 (12.7)	23.8 (9.5)	33.3 (13.3)	24.7 (10)	19.8 (7.9)	25.8 (10.3)	19.6 (7.9)	23.2 (9.3)	
Yellow	34.0 (13.6)	32.0 (12.8)	43.4 (17.4)	23.3 (9.3)	36.4 (14.6)	27.3 (10.9)	25.8 (10.3)	37.3 (14.9)	35.6 (14.2)	23.1 (9.2)	30.3 (12.3)	29.1 (11.7)	41.3 (16.5)	34.6 (13.9)	29.5 (11.8)	
Blue (Total)	64.0 (25.6)	64.0 (25.6)	88.0 (35.2)	59.6 (23.9)	70.6 (28.3)	60.4 (24.2)	81.8 (32.7)	60.7 (24.3)	69.7 (27.9)	69.1 (27.6)	74.0 (30.1)	64.8 (26)	74.7 (29.9)	61.7 (24.8)	82.6 (33.1)	
Blue (Provided)	52.0 (20.8)	35.0 (14)	75.8 (30.3)	45.7 (18.3)	58.1 (23.2)	43.8 (17.5)	70.8 (28.3)	51.5 (20.6)	53 (21.2)	62.9 (25.2)	74.0 (30.1)	63.5 (25.5)	58.0 (23.2)	61.5 (24.7)	78.4 (31.4)	
Blue (Rationed)	12.0 (4.8) [a](18.8)	29.0 (11.6) [a](45.3)	12.2 (4.9) [a](13.9)	13.9 (5.6) [a](23.4)	12.5 (5) [a](17.8)	16.6 (6.6) [a](27.8)	11.0 (4.4) [a](13.4)	9.2 (3.7) [a](15.2)	16.7 (6.7) [a](24.0)	6.2 (2.5) [a](9.0)	0.0 (0.0) [a](0.0)	1.3 (0.5) [a](1.9)	16.7 (6.7) [a](22.4)	0.2 (0.1) [a](0.4)	4.2 (1.7) [a](5.1)	161.9 [b]
Total Intubated	250	250	250	249.99	250	249.95	249.95	250	249.96	250	245.99	249.3	250	248.5	249.91	

The number of individuals n and (%) of all patients intubated for any day during the crisis period.

[a] The % of individuals selected for ventilator rationing out of all patients triaged as Blue.

[b] Does not include the two patients excluded from Step 1.

eTable 5: iNYVAG Survival & Demographic information for those triaged as Blue & Selected for Ventilator Reallocation, and those Receiving a Reallocated Ventilator

iNYVAG	Step 2: Resource Allocation	Step 3: Time Trial & Resource Reallocation					
	Ventilator Rationed n (%)			P Total Ventilators Rationed iNYVAG vs NYVAG	Receiving Rationed Ventilator n (%) [a]	P Receiving Rationed Ventilator iNYVAG vs NYVAG	p iNYVAG Rationed-Received
	Withheld	Withdrawn	Total				
Total	9.4 (5.0)	178.2 (95.0)	187.6 (100)	-	178.3 (100)	-	-
Survive [95%CI]	1.6 (17.2) [0-38.2]	45.8 (25.7) [22.5-28.9]	47.4 (25.3) [22.1-28.4]	**0.0002**	61.7 (34.6) [29.3-39.9]	0.94	0.051
SOFA	Figure e4			-	-	-	-
Age [95%CI]	63.3 [46.9-79.7]	64.2 [39.4-89.0]	64.2 [39.6-88.8]	0.27	64.3 [37.1-91.5]	0.89	0.94
Male	5.1 (53.8)	133.8 (75.0)	138.8 (73.9)	0.84	120.5 (67.6)	0.99	0.18
Female	4.3 (65.2)	44.4 (25.0)	48.8 (26.0)	0.84	57.7 (32.4)	0.99	0.18
Race							
African American	2.3 (24.8)	16.5 (9.2)	18.8 (10.0)	0.62	21.3 (11.9)	0.94	0.55
Asian	1.2 (13.0)	21.4 (11.9)	22.6 (12.0)	0.40	20.6 (11.5)	0.85	0.88
Native American	0 (0)	2.4 (1.3)	2.4 (1.2)	0.50	0.9 (0.4)	0.90	0.66
Unknown	2.7 (28.8)	61.3 (34.3)	64.0 (34.1)	0.47	60.5 (33.9)	0.97	0.97
White	3.1 (33.2)	76.7 (43.0)	79.9 (42.5)	0.30	75.0 (42.0)	0.85	0.93
Ethnicity							
Hispanic	2.3 (24.9)	46.2 (25.9)	49.1 (26.1)	0.41	46.9 (26.3)	0.95	0.98
Non-Hispanic	5.7 (60.2)	117.8 (66.0)	124.2 (66.1)	0.45	124.2 (66.1)	0.99	0.93
Unknown	1.4 (14.8)	14.3 (8.0)	14.4 (7.6)	0.99	14.4 (7.6)	0.93	0.83

95% Confidence Interval (CI), iNYVAG Rationed refers to Total Ventilators Rationed.

[a] Of those newly intubated during the crisis period who received a reallocated ventilator, 56.2% were Red, 35.5% were Yellow, and 6.1% were Blue. The total of ventilator rationed does not equal the total receiving reallocated ventilators because some individuals were front-end triaged not to receive a ventilator (rationed) but did not technically have a ventilator to provide to another individual.

© 2023 Walsh BC et al. *JAMA Network Open.*

eTable 6: Missing SOFA information for any day a patient was being assessed by NYVAG, the day of ventilator rationing, or the previous assessment to the day of ventilator rationing

NYVAG & iNYVAG	Any Day[a] a Patient could be Assessed by NYVAG	Assessment on the Day of Ventilator Rationing[b]	Previous Assessment to Day of Ventilator Reallocation[b]	Potential NYVAG Assessment Day Vs. Assessment on the Day of Ventilator Rationing	Potential NYVAG Assessment Day Vs. Previous Assessment to Day of Ventilator Reallocation	Assessment on the Day of Ventilator Rationing Vs. Previous Assessment to Day of Ventilator Reallocation
	Patient-SOFA-Days n (%)			p value		
Total	2364 (100)	[c] 161.6 (100)	[d] 154.1 (100)	-	-	-
Anything Missing	1921 (81.3)	124.2 (76.8)	129.1 (84.3)	0.17	0.44	0.12
Cardiovascular	92 (3.9)	3.9 (2.4)	4.7 (3.1)	0.34	0.60	0.72
Coagulation	130 (5.5)	4.8 (2.9)	14.9 (9.7)	0.17	**0.03**	**0.01**
Hepatic	748 (31.6)	46.7 (28.9)	46.6 (30.4)	0.47	0.72	0.79
Nephrology	59 (2.5)	0.7 (0.4)	5.5 (3.6)	0.10	0.42	**0.04**
Neurology	1659 (70.2)	100.9 (62.3)	114.1 (74.5)	**0.04**	0.31	**0.03**
Pulmonary	23 (1.0)	1.5 (1.0)	4.6 (3.0)	0.96	**0.02**	0.19
Missing # of Categories						
0	443 (18.7)	37.6 (23.3)	24.0 (15.7)	0.16	0.33	0.08
1	1340 (56.7)	95.4 (59.0)	87.3 (57.0)	0.56	0.99	0.68
2	455 (19.2)	24.4 (15.1)	28.4 (18.6)	0.19	0.80	0.42
3	73 (3.1)	4.1 (2.5)	8.7 (5.7)	0.69	0.08	0.16
4	32 (1.4)	0.062 (0.04)	3.5 (2.3)	0.15	0.35	0.06
5	17 (0.7)	0 (0)	1.0 (0.7)	-	0.92	-
**6 (Everything)**	**16 (0.7)**	**0 (0)**	**0 (0)**	-	-	-

[a] As observed in the real world during the crisis period.
[b] Averaged within each simulation, and then averaged across all simulations.
[c] Excludes the 2 patients excluded from NYVAG Step 1.
[d] Excludes the 9.6 individuals who had their ventilator withheld on the day of consideration for intubation, and consequently do not have a previous SOFA score that was used as a comparison.

© 2023 Walsh BC et al. *JAMA Network Open.*

eTable 7: Missing SOFA scores, including subcategories, for Patient's Triaged on the day of Consideration for Intubation during the crisis period.

	Triage of Patients on Day of Consideration for Intubation (Step 2)					
	**Red** SOFA < 7 n (%)		**Yellow** SOFA 8-11 n (%)		**Blue** SOFA > 11 n (%)	
Total Patients	244 (100)		161 (100)		49 (100)	
Survive to Discharge	98 (40.2)		50 (31.0)		11 (22.5)	
Anything Missing	231 (94.7)		136 (85.5)		35 (71.4)	
Cardiovascular	45 (18.4)		4 (2.5)		1 (2.0)	
Coagulation	70 (28.7)		12 (7.5)		5 (10.2)	
Hepatic	109 (44.7)		39 (24.2)		17 (34.7)	
Pulmonary	17 (7.0)		0 (0)		1 (2.0)	
Nephrology	42 (17.2)		5 (3.1)		1 (2.0)	
Neurology	217 (88.9)		121 (75.2)		21 (42.9)	
Everything	10 (4.1)		1 (0.6)		5 (10.2)	
Number of Missing SOFA Subcategories	Number of Patients Missing the Corresponding Number of Subcategories n (%)					
	Total	Survive to Discharge	Total	Survive to Discharge	Total	Survive to Discharge
0 (None)	13 (5.3)	6 (46.2)	23 (14.3)	11 (47.8)	14 (28.8)	5 (37.7)
1	98 (40.2)	53 (53.5)	104 (64.6)	32 (30.8)	26 (53.1)	5 (19.2)
2	58 (23.8)	24 (40.0)	26 (14.1)	6 (23.1)	4 (8.2)	0 (0)
3	26 (10.7)	4 (14.8)	6 (3.7)	1 (16.7)	0 (0)	-
4	24 (9.8)	7 (28.0)	1 (0.6)	0 (0)	0 (0)	-
5	15 (6.1)	4 (26.7)	0 (0)	-	0 (0)	-
6 (Everything)	10 (4.1)	0 (0)	1 (0.6)	0 (0)	5 (10.2)	1 (20.0)
0 - 2	169 (69.3)	83 (49.1)	153 (95.0)	49 (32.0)	44 (89.8)	10 (22.7)
3 - 6	75 (30.7)	15 (20)	8 (5.0)	1 (12.5)	5 (10.2)	1 (20.0)

© 2023 Walsh BC et al. *JAMA Network Open.*

eTable 8: Characteristics of individuals selected for ventilator rationing that occurred for all iNYVAG simulations

iNYVAG[100%]	Ventilator Rationed n (%)	p value iNYVAG[100%] vs NYVAG
Total	81 (100)	-
Survive	11 (13.6)	< 0.000001
Age [95%CI]	62.8 [35.8-89.8]	0.96
Male	61 (75.3)	0.70
Female	20 (24.7)	
Race		
African American	6 (7.4)	0.30
Asian	12 (14.8)	0.19
Native American	0 (0)	-
Unknown	32 (39.5)	0.82
White	31 (38.3)	0.15
Ethnicity		
Hispanic	23 (28.4)	0.30
Non-Hispanic	50 (61.7)	0.20
Unknown	8 (9.9)	0.55

© 2023 Walsh BC et al. *JAMA Network Open.*

eFigure 1: NYVAG Step 1 Exclusionary Criteria [1]

Step 1 - List of Exclusion Criteria for Adult Patients
Medical Conditions that Result in Immediate or Near-Immediate Mortality
Even with Aggressive Therapy

1) Cardiac arrest: unwitnessed arrest, recurrent arrest without hemodynamic stability, arrest unresponsive to standard interventions and measures; trauma-related arrest
2) Irreversible age-specific hypotension unresponsive to fluid resuscitation and vasopressor therapy
3) Traumatic brain injury with no motor response to painful stimulus (i.e., best motor response = 1) (See Appendix 1)
4) Severe burns: where predicted survival ≤ 10% even with unlimited aggressive therapy (See Appendix 1)
5) Any other conditions resulting in immediate or near-immediate mortality even with aggressive therapy[1]

[1] This “catch all” phrase encompasses other possibilities because the list above is merely a guide and does not list every medical condition that would result in immediate or near-immediate mortality.

[1] New York Ventilator Allocation Guidelines. New York State Task Force on Life and the Law & New York State Department of Health. https://www.health.ny. gov/regulations/task_force/reports_publications/docs/ventilator_guidelines. pdf. Published November 2015. Accessed January 13, 2021.

© 2023 Walsh BC et al. *JAMA Network Open.*

eFigure 2: Classification of Blue Patients Selected have their Ventilator Rationed with their Observed Survival to Discharge (NYVAG)

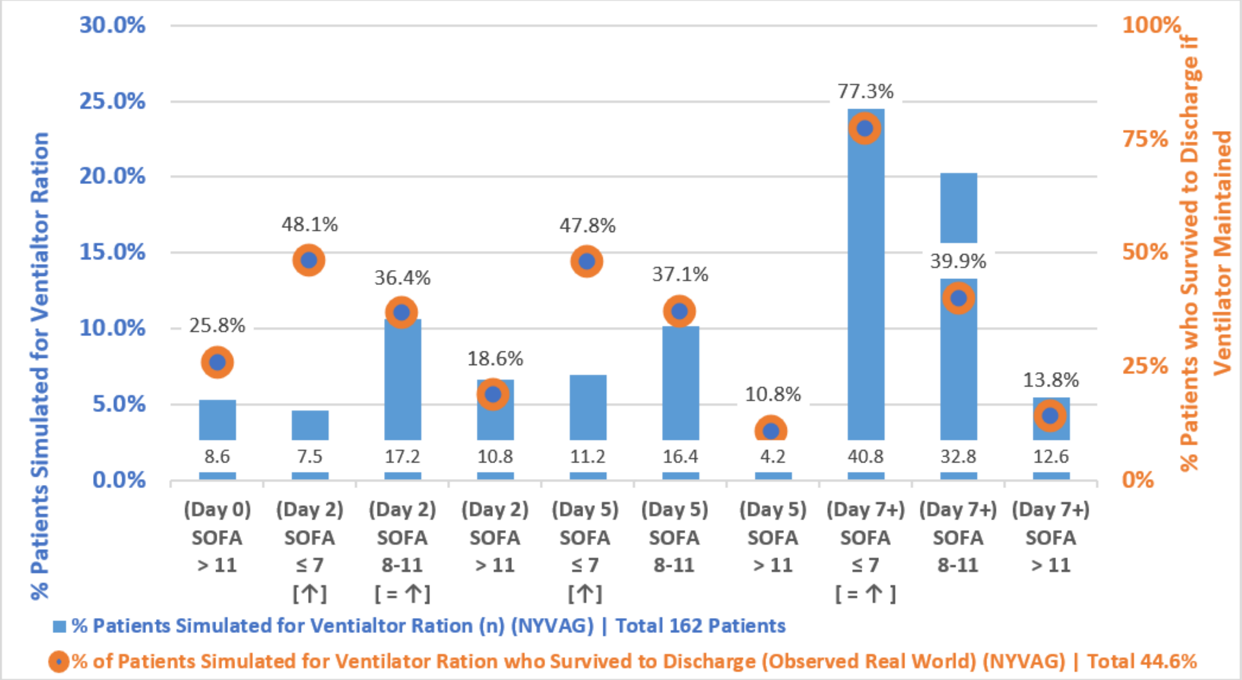

Patients triaged as Blue and randomly chosen to have their ventilator rationed by day of ventilation (parenthesis), SOFA score on the day of ventilator rationing, and dynamic comparison to previous SOFA assessment [brackets].

© 2023 Walsh BC et al. *JAMA Network Open.*

eFigure 3: Observed Survival to Discharge by Blue Subcategorization for NYVAG & iNYVAG

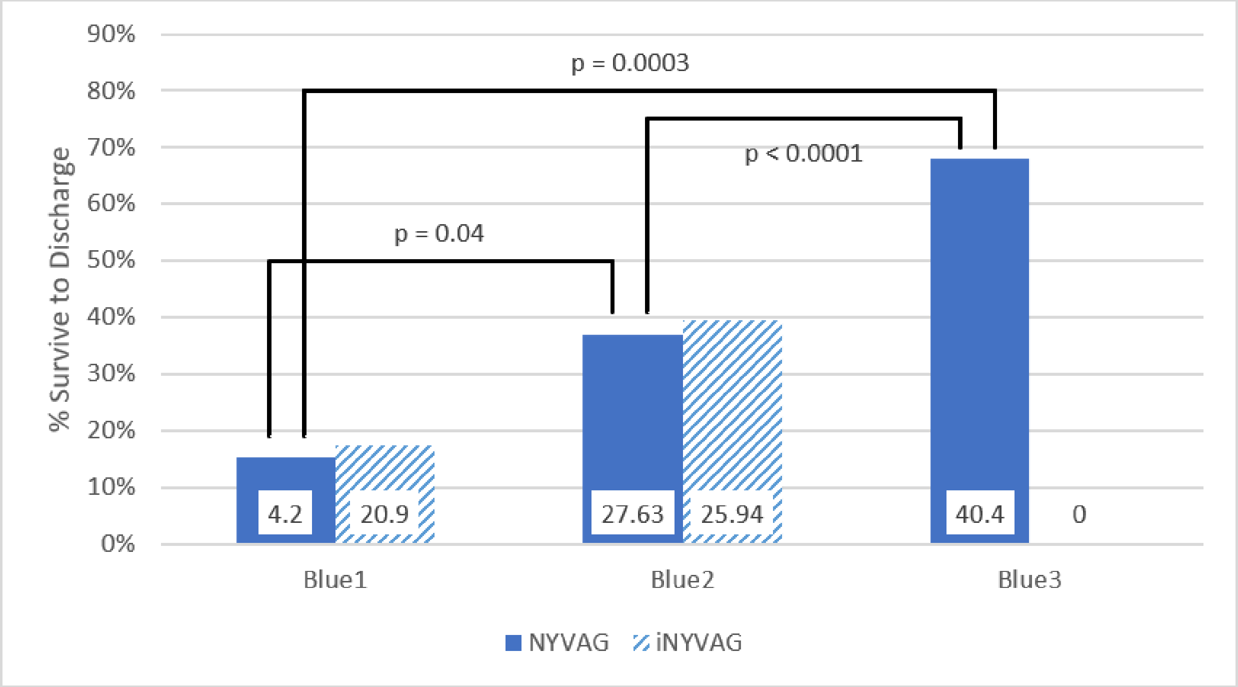

© 2023 Walsh BC et al. *JAMA Network Open.*

eFigure 4: Classification of Blue Patients Selected have their Ventilator Rationed with their Observed Survival to Discharge (NYVAG vs iNYVAG)

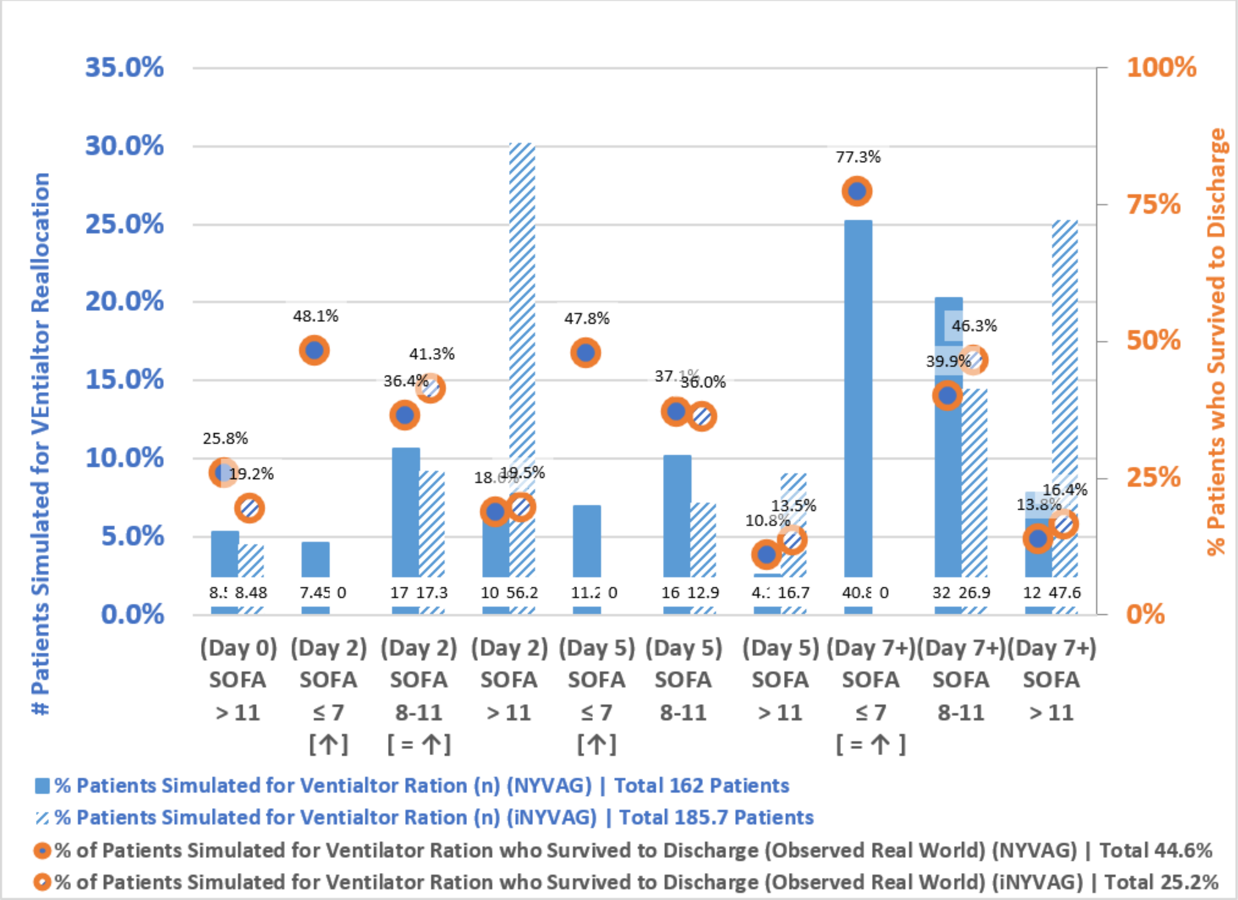

Solid and dashed represent NYVAG & iNYVAG respectively. Patients triaged as Blue and randomly chosen to have their ventilator rationed by day of ventilation (parenthesis), SOFA score on the day of ventilator rationing, and dynamic comparison to previous SOFA assessment [brackets].

© 2023 Walsh BC et al. *JAMA Network Open.*

eFigure 5: How often the same-individual was selected for ventilator rationing across all simulations by triage strategy

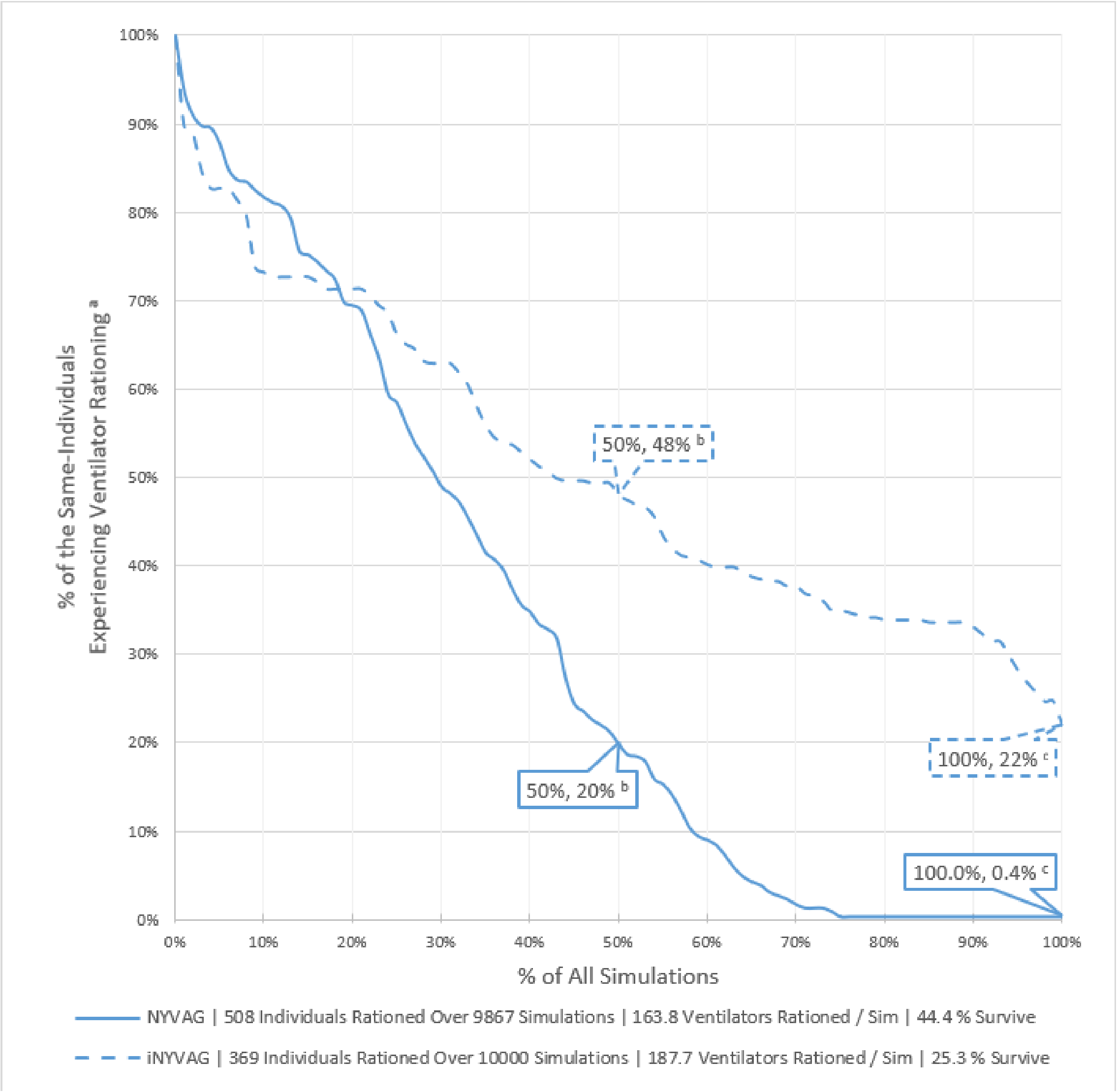

[a] Y-axis represents the % of the same-individual who experienced ventilator rationing, out of all individuals that experienced ventilator rationing in 10,000 simulations.

[b] 50% of all simulations resulted in the same 20% (n=102) and 48% (n=177) individuals experienced ventilator rationing in NYVAG vs. iNYVAG respectively.

[c] 100% of all simulations resulted in the same 0.4% (n=2) and 22% (n=81) individuals experienced ventilator rationing in NYVAG vs. iNYVAG respectively.

© 2023 Walsh BC et al. *JAMA Network Open.*
