# Supplementary material for: Simulation of New York City’s Ventilator Allocation Guideline During the Spring 2020 COVID-19 Surge
Source: JAMA Netw Open. 2023 Oct 5;6(10):e2336736. doi: 10.1001/jamanetworkopen.2023.36736 (PMC10556967; doi:10.1001/jamanetworkopen.2023.36736)
Supplement: Supplement 2. — Data Sharing Statement [file jamanetwopen-e2336736-s002.pdf]

Data Sharing Statement

Walsh. Simulation of New York City's Ventilator Allocation Guideline During the Spring 2020 COVID-19 Surge. *JAMA Netw Open*. Published October 03, 2023. doi:10.1001/jamanetworkopen.2023.36736

Data
Data available: No

Additional Information
Explanation for why data not available: Our IRB approval does not include disseminating de-identifying to individuals outside the research group.
